# Clinical correlates, procedural benchmarks, and reperfusion–outcome dissociation after mechanical thrombectomy for acute large-vessel occlusion stroke: a single-center retrospective registry study

**DOI:** 10.3389/fneur.2026.1835315

**Published:** 2026-07-30

**Authors:** Hao Xu, Zhouming Ren, Xinzheng Fu, Xuyan Zhang

**Affiliations:** Department of Neurology, Haining People's Hospital, Jiaxing, Zhejiang, China

**Keywords:** functional independence, large-vessel occlusion, logistic regression, mechanical thrombectomy, modified Rankin Scale, retrospective registry

## Abstract

**Background:**

Endovascular thrombectomy (EVT) is an established treatment for selected patients with acute ischemic stroke caused by large-vessel occlusion (LVO). Single-center registries remain useful for reviewing outcomes, safety events, and workflow in routine care provided that their analytic limits are clearly stated.

**Methods:**

We performed a retrospective analysis of a single-center mechanical thrombectomy registry covering January 2023 to December 2025. The final cohort included 212 patients; 90-day modified Rankin Scale (mRS) data were available in 206 (97.2%). The primary outcome was functional independence at 90 days (mRS 0–2). Procedural and safety outcomes were summarized for the full cohort. Two pre-specified logistic regression models were used to explore clinical and procedural correlates and were interpreted with explicit caution regarding sample size and events-per-variable limitations. Robustness was assessed using (i) Firth penalized maximum-likelihood estimation, (ii) an anterior-circulation-only sensitivity Model 1, (iii) E-value computation and inverse-probability-of-treatment weighting for the intravenous thrombolysis (IVT) association, (iv) four-scenario handling of missing 90-day mRS including multiple imputation by chained equations, (v) optimism-corrected area under the curve (AUC) with 2,000-iteration bootstrap, and (vi) a *post-hoc* Model 1 incorporating collateral circulation score.

**Results:**

Among patients with available 90-day follow-up, 111 of 206 (53.9%) achieved functional independence; across alternative handling of the 6 patients with missing follow-up, the proportion ranged from 52.4% (worst-case) to 55.2% (best-case), with multiple imputation yielding 53.3%. Baseline NIHSS score showed the most consistent adjusted association with lower odds of functional independence [Model 1: adjusted odds ratio (aOR) 0.95, 95% CI 0.91–0.99; Firth aOR 0.95, 95% CI 0.91–0.99]. Intravenous thrombolysis before thrombectomy showed a numerical adjusted association with functional independence (Model 1 aOR 2.14, 95% CI 1.11–4.13; Firth aOR 2.04, 95% CI 1.09–3.91). This magnitude is much larger than randomized-trial pooled estimates (relative risk approximately 1.05–1.15) and is best explained by confounding by indication. The E-value of 2.10 for the point estimate (1.29 for the lower confidence bound) indicates that a moderately strong unmeasured confounder could explain the observed association. In an anterior-circulation-only sensitivity analysis (*n* = 171), the IVT association no longer reached statistical significance (aOR 2.02, 95% CI 0.98–4.17), reflecting both reduced statistical power and territory-dependent case mix. Overlap weighting, which achieved exact balance on all measured propensity-score covariates whereas standard inverse-probability-of-treatment weighting did not (residual onset-to-door SMD = 0.74), produced an attenuated IVT aOR of 1.88 (95% CI 0.58–6.09)—no longer statistically significant and close to the pooled randomized-trial effect size. No workflow or procedural variable reached statistical significance after adjustment. Apparent discrimination was moderate (C-statistic: Model 1, 0.679; Model 2, 0.703); optimism-corrected AUC was 0.60 for both models, the expected pattern of overfitting in low-EPV settings. Despite a TICI 2b−3 reperfusion rate of 99.5%, 45.9% of reperfused patients failed to achieve functional independence—a *reperfusion–outcome gap* that mirrors the increasingly recognized phenomenon of clinically ineffective reperfusion.

**Conclusion:**

In this single-center retrospective registry, baseline stroke severity was the most consistent clinical correlate of 90-day functional independence after mechanical thrombectomy, holding up across Firth-penalized, anterior-circulation-only, and collateral-adjusted sensitivity analyses. The IVT association lost statistical significance under overlap weighting (aOR 1.88, 95% CI 0.58–6.09), which we read as confounded rather than causal and which brings the present registry in line with the small randomized-trial effect size. Procedural success was exceptionally high yet decoupled from functional recovery in nearly half of patients. The data are best viewed as a local EVT benchmark and as a basis for quality review, including continued attention to door-to-EVT workflow and to mechanisms of futile reperfusion.

## Introduction

Acute ischemic stroke caused by large-vessel occlusion (LVO) is a major cause of death and long-term disability (17, 18). Current American Heart Association/American Stroke Association guidelines (Class I recommendation) endorse mechanical thrombectomy for eligible anterior-circulation LVO patients within 24 h of last known well when imaging supports salvageable tissue (19), and recent evidence has extended consideration to selected posterior-circulation and large-core presentations (5, 6). In this pathway, both patient factors at presentation and treatment workflow can influence recovery (20).

Randomized trials and pooled analyses have shown that endovascular treatment improves functional outcomes in selected patients with anterior-circulation LVO (1–4, 21). Later studies extended eligibility to selected patients treated in longer time windows when imaging suggested salvageable tissue (5, 6). Trial populations and everyday practice are not identical, though. Local registries therefore remain useful because they show how outcomes, safety events, reperfusion rates, and workflow times appear in routine hospital settings (22, 23).

We do not present National Institutes of Health Stroke Scale (NIHSS) or thrombolysis as new biological discoveries. Baseline stroke severity has been repeatedly linked to endovascular thrombectomy (EVT) outcome in pooled trial data (24) and in large registries (25), and the value of bridging intravenous thrombolysis (IVT) before EVT has been examined in randomized non-inferiority trials (9, 10) and individual-patient-data meta-analyses (11, 12). The value of the present study is more practical. It describes a consecutive EVT experience from a regional Chinese stroke center during 2023–2025, covering 90-day mRS outcomes, reperfusion performance, hemorrhage and mortality rates, and workflow times. These data may help local quality review and provide a transparent comparison point for other centers, while avoiding claims that exceed the design.

We therefore aimed to describe clinical outcomes, procedural performance, and safety results after mechanical thrombectomy for acute LVO stroke at Haining People's Hospital, and to examine clinical and workflow correlates of 90-day functional independence as exploratory registry findings.

## Methods

### Study design and setting

We performed a retrospective analysis of a single-center mechanical thrombectomy registry at Haining People's Hospital, Haining, Jiaxing, Zhejiang, China. The study period covered January 1, 2023, to December 31, 2025. The report follows the Strengthening the Reporting of Observational Studies in Epidemiology (STROBE) statement (26).

### Patient identification and selection

**Figure 1:**
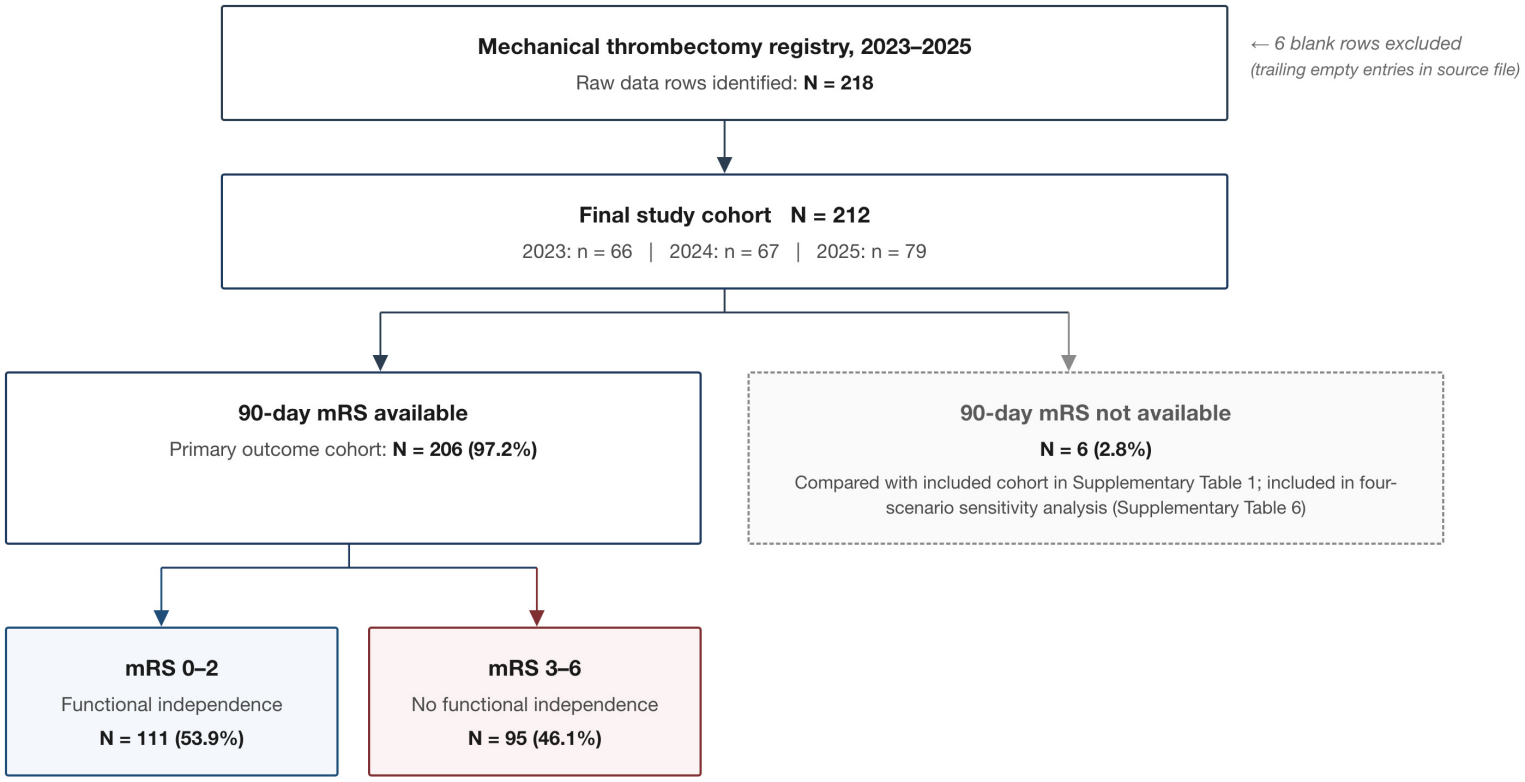
Study flowchart. The registry source file contained 218 data rows; six blank rows with no patient data were removed, yielding a final cohort of 212 patients. Of these, 206 (97.2%) had available 90-day modified Rankin Scale (mRS) data and formed the complete-case primary outcome cohort. The six patients without 90-day follow-up are described separately. Across alternative handling of missing 90-day mRS (complete-case, best-case, worst-case, multiple imputation by chained equations), the functional-independence proportion ranged from 52.4 to 55.2%. The denominator for procedural and safety outcomes was *N* = 212. Analytic sample sizes for downstream analyses: descriptive procedural and safety outcomes are summarized for the full cohort (*N* = 212); the complete-case primary outcome analysis is restricted to patients with available 90-day mRS (*n* = 206); Model 1 includes patients with complete baseline covariates (*n* = 204); Model 2 additionally requires workflow variables (*n* = 165); the anterior-circulation-only sensitivity Model 1 is restricted to ICA, MCA, and tandem-ICA-MCA cases (*n* = 171); and the four-scenario sensitivity analysis for the 6 patients without 90-day mRS yields functional-independence proportions ranging from 52.4% (worst-case) to 55.2% (best-case), with multiple imputation by chained equations pooling to 53.3%. mRS, modified Rankin Scale; ICA, internal carotid artery; MCA, middle cerebral artery.

Patients were identified from the departmental mechanical thrombectomy registry. Eligibility criteria were: age 18 years or older; acute ischemic stroke with angiographically confirmed LVO of the anterior or posterior circulation; mechanical thrombectomy performed at Haining People's Hospital during the study period; and available baseline neurological assessment at presentation. The registry source file contained 218 rows; after six blank rows without patient information were removed, the final cohort included 212 patients (Figure 1). Patients without 90-day mRS data (*n* = 6, 2.8%) were retained for descriptive and safety analyses and were compared with patients who had follow-up data.

Pre-stroke modified Rankin Scale was not systematically documented in the registry; this limitation is acknowledged explicitly in the Discussion. The closest available proxy in the dataset is documented history of prior stroke or transient ischemic attack (binary), which we used in a *post-hoc* Model 1 sensitivity analysis (Supplementary Table 10) to partially address this gap. We did not impute pre-stroke mRS values that could not be verified.

### Data collection and variable definitions

Data were collected retrospectively from clinical records, neuroimaging reports, and procedural documentation. Variables included demographics, vascular risk factors, admission clinical parameters, baseline National Institutes of Health Stroke Scale (NIHSS) score, admission Alberta Stroke Program Early CT Score (ASPECTS), responsible vessel territory, intravenous thrombolysis before thrombectomy, workflow timing metrics, number of thrombectomy passes, recanalization technique, final Thrombolysis in Cerebral Infarction (TICI) reperfusion grade (27), and pre-procedural collateral circulation graded on the Higashida 0–4 computed tomography angiography (CTA) collateral score (27).

Baseline NIHSS was assessed by the treating team before the procedure. Intravenous thrombolysis before thrombectomy was defined as administration of intravenous alteplase before or concurrent with thrombectomy, regardless of whether it was administered at the referring or treating hospital (21); urokinase was not classified as intravenous thrombolysis for this purpose. One patient had an uncodeable thrombolysis entry and was treated as missing. Door-to-puncture time was calculated from emergency department arrival to femoral artery puncture; patients with in-hospital stroke onset were excluded from this calculation (28). Successful reperfusion was defined as TICI 2b−3 (27). Vessel territory was classified as internal carotid artery (ICA), middle cerebral artery (MCA), tandem ICA+MCA co-occlusion, basilar artery, or other. Sub-segment-level information (M1 vs. M2 within the MCA territory; petrous, cavernous, or terminus within the ICA; proximal vs. mid-basilar) was not consistently coded in the registry export and could therefore not be analyzed separately, despite being available in the source digital subtraction angiography reports—a limitation acknowledged below.

ASPECTS was recorded as available in the source registry (29). Conventional ASPECTS is primarily an anterior-circulation score. Because posterior-circulation-specific scores such as pc-ASPECTS were not systematically available, ASPECTS results are reported for transparency but interpreted cautiously, and are not presented as a general imaging predictor across all vessel territories. To address this directly, we pre-specified an anterior-circulation-only sensitivity analysis (see Statistical analysis).

### Outcome definitions

The primary outcome was 90-day functional independence, defined as an mRS score of 0–2 (30). At the study institution, 90-day follow-up is conducted through outpatient clinic review and structured telephone assessment depending on patient availability and mobility. Secondary outcomes included the ordinal 90-day mRS distribution, successful reperfusion, any intracranial hemorrhage on post-procedural neuroimaging (31), in-hospital mortality, NIHSS at 24 h, change in NIHSS from baseline to 24 h, and length of hospital stay. Any intracranial hemorrhage was defined as any hemorrhagic event documented in the source registry; symptomatic and asymptomatic events could not be distinguished. In-hospital death was ascertained from discharge records using explicit death-related endpoint terms; registry entries in the mRS field coded as death terms were recoded to mRS = 6 for outcome analyses.

### Statistical analysis

Continuous variables are reported as mean ± standard deviation or median [interquartile range (IQR)] as appropriate. Categorical variables are presented as number and percentage. Between-group comparisons used Student's *t*-test or the Mann-Whitney *U*-test for continuous variables and chi-square or Fisher's exact test for categorical variables, as appropriate. All analyses were performed in Python 3.9 (Python Software Foundation, Wilmington, DE, United States) using *pandas, statsmodels* (32), *scikit-learn* (33), and *firthlogist*.

The primary regression analysis was a complete-case binary logistic regression restricted to patients with available 90-day mRS. Model 1 included pre-specified baseline variables entered simultaneously without stepwise selection: age, sex, baseline NIHSS score, baseline ASPECTS, atrial fibrillation, diabetes mellitus, hypertension, intravenous thrombolysis before thrombectomy, and vessel territory (MCA reference). Model 2 added door-to-puncture time, puncture-to-recanalization time, and number of thrombectomy passes.

Because the events-per-variable ratio in both models was below the conventional threshold of 10, we conducted a pre-specified robustness analysis using Firth's penalized maximum-likelihood estimation (7), which reduces small-sample bias and is recommended for low-events per variable (EPV) logistic regression. Firth-corrected adjusted odds ratios with profile-penalized-likelihood 95% confidence intervals are reported alongside conventional maximum-likelihood estimates (Supplementary Table 3). Four additional sensitivity analyses were pre-specified:

1. Anterior-circulation-only Model 1 (Supplementary Table 4), restricted to ICA, MCA, and tandem-ICA+MCA cases (*n* = 171; events = 90), to address the limitation that conventional ASPECTS is not validated in posterior-circulation stroke and that 16% of the cohort had non-anterior occlusions.
2. Intravenous thrombolysis robustness (Supplementary Table 5): the E-value (8) was computed for the IVT adjusted odds ratio after conversion to an approximate adjusted relative risk using the baseline outcome rate in the unexposed group. Two complementary propensity-score (PS) adjustments were performed using a PS model that included age, sex, baseline NIHSS, ASPECTS, atrial fibrillation, diabetes, hypertension, and onset-to-door time: (i) standard inverse-probability-of-treatment weighting (IPTW) with weights truncated at the 1st and 99th percentile; and (ii) overlap weighting (13), which weights each patient by the propensity that they would have received the opposite treatment and is known to achieve exact balance on the variables used to estimate the propensity score. Covariate balance after weighting was quantified by standardized mean differences (SMDs); an SMD with absolute value < 0.10 is conventionally regarded as acceptable balance. Both weighted analyses were used to estimate the IVT effect on functional independence within the same Model 1 covariate set.
3. Missing 90-day mRS handling (Supplementary Table 6): four scenarios were compared for the descriptive functional-independence proportion—complete-case, best-case (all six missing classified as independent), worst-case (all six classified as poor outcome), and multiple imputation by chained equations (MICE, 20 imputations) using *statsmodels.imputation.mice*.
4. Collateral circulation as additional covariate (Supplementary Table 8): a *post-hoc* Model 1 incorporated the 0–4 pre-procedural collateral score available in 211 of 212 patients to evaluate whether the NIHSS and IVT associations were sensitive to collateral status.

Model discrimination was reported as the apparent C-statistic and, alongside it, the optimism-corrected C-statistic using 2,000-iteration bootstrap resampling (Harrell-style correction). Apparent calibration was summarized by visual inspection of the calibration curve (Supplementary Figure 1).

ASPECTS was retained in the pre-specified models for transparency because it was captured in the registry and included in the original analysis plan. However, because conventional ASPECTS is not designed for posterior-circulation stroke, the ASPECTS coefficient is interpreted cautiously and is supported by the anterior-circulation-only sensitivity analysis described above.

## Results

### Patient flow and cohort characteristics

The final study cohort comprised 212 patients (2023: *n* = 66; 2024: *n* = 67; 2025: *n* = 79). Of these, 206 (97.2%) had 90-day mRS data and formed the complete-case primary outcome cohort. Among patients with available follow-up, 111 of 206 (53.9%) achieved functional independence. Across alternative handling of the six patients with missing follow-up, the proportion ranged from 111 of 212 (52.4%, worst-case) to 117 of 212 (55.2%, best-case), with multiple imputation yielding 53.3% (range across imputations: 52.4–54.2%; Supplementary Table 6).

The six patients without 90-day follow-up had higher baseline NIHSS scores (median 25.5 vs. 19.0; *p* = 0.037) and higher in-hospital mortality [3/6 (50.0%) vs. 12/206 (5.8%); *p* = 0.005] than patients with available follow-up. These differences suggest that follow-up was unlikely to be missing completely at random and that the complete-case estimate may slightly overstate the rate of functional independence. The worst-case sensitivity bound (52.4%) provides a conservative lower limit, and the MICE estimate (53.3%)—which leverages the strong baseline-NIHSS and in-hospital-death predictors for the missing observations—tracks the complete-case rate closely.

### Baseline characteristics

Baseline characteristics are summarized in Table 1 with denominators restricted to the 206-patient outcome cohort (Supplementary Table 1 separately compares included and excluded patients). Mean age was 70.5 ± 12.1 years, and 124 patients (60.2%) were male. Median baseline NIHSS score was 19 (IQR 12–25), reflecting a predominantly severe stroke cohort. Median ASPECTS was 9 (IQR 9–10), indicating a narrow distribution. Median pre-procedural collateral score was 2 (IQR 1–3). The most frequently affected territories were MCA (107 patients, 51.9%) and ICA (56, 27.2%). Intravenous thrombolysis before thrombectomy was administered in 64 of 205 patients with available thrombolysis data in the outcome cohort (31.2%).

**Table 1:** Baseline demographic, clinical, imaging, and workflow characteristics of the outcome cohort (N = 206).

Variable	Overall (*N* = 206)	mRS 0–2 (*n* = 111)	mRS 3–6 (*n* = 95)	*p*-Value
Age, years, mean ± SD	70.5 ± 12.1	69.3 ± 12.6	72.0 ± 11.4	0.104
Male sex, *n* (%)	124 (60.2)	68 (61.3)	56 (58.9)	0.845
Body mass index, kg/m^2^, mean ± SD	23.9 ± 3.5	23.9 ± 3.4	24.0 ± 3.7	0.770
Hypertension, *n* (%)	134 (65.0)	67 (60.4)	67 (70.5)	0.168
Diabetes mellitus, *n* (%)	50 (24.4)	28 (25.5)	22 (23.2)	0.827
Atrial fibrillation, *n* (%)	77 (37.4)	36 (32.4)	41 (43.2)	0.149
Current smoking, *n* (%)	33 (16.0)	22 (19.8)	11 (11.6)	0.156
Prior stroke/TIA, *n* (%)	24 (11.7)	12 (10.8)	12 (12.6)	0.682
Coronary artery disease, *n* (%)	47 (22.8)	24 (21.6)	23 (24.2)	0.654
Systolic BP, mmHg, mean ± SD	153.2 ± 23.8	152.4 ± 22.8	154.1 ± 25.1	0.619
Diastolic BP, mmHg, mean ± SD	84.5 ± 13.2	83.1 ± 12.3	86.1 ± 14.0	0.108
Blood glucose, mmol/L, median (IQR)	7.0 (5.8–9.5)	6.8 (5.7–9.4)	7.0 (5.9–9.6)	0.467
Serum creatinine, μmol/L, median (IQR)	75.0 (62.0–89.8)	72.0 (59.0–84.0)	76.0 (65.0–96.0)	0.283
Baseline NIHSS, median (IQR)	19.0 (12.0–25.0)	18.0 (11.0–24.0)	20.0 (14.5–26.0)	**0.025**
Baseline ASPECTS, median (IQR)	9.0 (9.0–10.0)	10.0 (9.0–10.0)	9.0 (8.0–10.0)	0.299
Higashida CTA collateral score (0–4) (27), median (IQR)	2.0 (1.0–3.0)	2.0 (1.0–3.0)	2.0 (1.0–3.0)	0.970
ICA territory, *n* (%)	56 (27.2)	30 (27.0)	26 (27.4)	
MCA territory, *n* (%)	107 (51.9)	56 (50.5)	51 (53.7)	
Tandem ICA+MCA, *n* (%)	10 (4.9)	5 (4.5)	5 (5.3)	
Basilar artery, *n* (%)	22 (10.7)	12 (10.8)	10 (10.5)	
Other territory, *n* (%)	11 (5.3)	8 (7.2)	3 (3.2)	0.783
Onset-to-door time, min, median (IQR)	152.0 (68.2–573.5)	144.0 (68.0–380.0)	190.0 (69.0–658.0)	0.493
Door-to-imaging time, min, median (IQR)	19.0 (15.0–24.0)	18.5 (15.8–24.0)	19.0 (15.0–23.0)	0.777
Door-to-puncture time, min, median (IQR)	98.5 (83.0–120.0)	98.0 (82.5–120.0)	99.0 (87.0–119.0)	0.795
IV thrombolysis before thrombectomy, *n* (%)	64 (31.2)	42 (37.8)	22 (23.4)	**0.038**

Values are mean ± SD, median (IQR), or n (%). All denominators correspond to the 206-patient outcome cohort (the 6 patients without 90-day mRS are characterized separately in Supplementary Table 1). Vessel-territory frequencies sum to 206. Conventional ASPECTS should be interpreted cautiously because posterior-circulation cases were included; an anterior-circulation-only sensitivity Model 1 is presented in Supplementary Table 4. IV thrombolysis denominators reflect the 205 outcome-cohort patients with coded thrombolysis status (one uncodeable entry was treated as missing). Bold indicates p < 0.05.

Compared with patients who did not achieve functional independence, those with mRS 0–2 had lower baseline NIHSS scores (median 18 vs. 20; *p* = 0.025). Intravenous thrombolysis before thrombectomy was more frequent among patients with functional independence [42/111 (37.8%) vs. 22/94 (23.4%); *p* = 0.038]. Other baseline variables, including collateral score (mean 1.80 vs. 1.80; *p* = 0.97), did not differ significantly between outcome groups. Exploratory univariable and multivariable logistic regression estimates are presented in Table 2.

**Table 2:** Exploratory logistic regression analyses for 90-day functional independence (mRS 0–2): full-cohort Model 1 and Model 2.

Variable	Univariable OR (95% CI)	*p*-Value	Model 1 aOR (95% CI)	*p*-Value	Model 2 aOR (95% CI)	*p*-Value
Age (per year)	0.98 (0.96–1.00)	0.108	0.99 (0.96–1.02)	0.511	0.99 (0.96–1.02)	0.653
Male sex	1.10 (0.63–1.93)	0.735	0.95 (0.51–1.77)	0.877	0.89 (0.43–1.82)	0.743
Baseline NIHSS (per point)	0.96 (0.92–0.99)	**0.021**	0.95 (0.91–0.99)	**0.008**	0.93 (0.89–0.98)	**0.004**
Baseline ASPECTS (per point)	1.10 (0.91–1.33)	0.314	1.09 (0.90–1.33)	0.385	1.11 (0.90–1.37)	0.332
Atrial fibrillation	0.63 (0.36–1.12)	0.114	0.87 (0.46–1.67)	0.679	0.80 (0.37–1.70)	0.554
Diabetes mellitus	1.13 (0.60–2.15)	0.703	1.65 (0.81–3.36)	0.169	1.16 (0.51–2.64)	0.722
Hypertension	0.64 (0.36–1.14)	0.128	0.58 (0.30–1.10)	0.096	0.52 (0.25–1.08)	0.079
IV thrombolysis before thrombectomy	1.99 (1.08–3.68)	**0.027**	2.14 (1.11–4.13)	**0.024**	2.47 (1.17–5.21)	**0.018**
ICA vs. MCA	0.98 (0.53–1.82)	0.956	1.39 (0.70–2.78)	0.350	0.93 (0.42–2.04)	0.855
Tandem ICA+MCA vs. MCA	0.85 (0.24–3.03)	0.801	1.04 (0.27–4.06)	0.956	0.79 (0.17–3.59)	0.762
Basilar artery vs. MCA	1.03 (0.42–2.50)	0.947	1.46 (0.53–4.00)	0.461	1.02 (0.33–3.19)	0.972
Other territory vs. MCA	3.61 (0.75–17.45)	0.110	2.11 (0.49–9.00)	0.315	2.22 (0.33–14.77)	0.411
Door-to-puncture time (per min)	—	—	—	—	0.995 (0.99–1.00)	0.243
Puncture-to-recanalization time (per min)	—	—	—	—	0.996 (0.99–1.00)	0.243
Number of thrombectomy passes	0.94 (0.71–1.26)	0.679	—	—	1.00 (0.66–1.50)	0.988
*N* (complete cases)	206		204		165	
Events (mRS 0–2)	111		110		86	
C-statistic, apparent (95% CI)	0.592 (0.514–0.665)		0.679 (0.606–0.751)		0.703 (0.632–0.787)	
C-statistic, optimism-corrected	0.590		0.601		0.600	

Model 1 included pre-specified baseline clinical and imaging variables entered simultaneously without stepwise selection. Model 2 additionally included workflow/procedural variables and had a smaller complete-case sample because of missing door-to-puncture time. Both models are exploratory association analyses, not validated prediction models; the substantial optimism (0.08–0.10) confirms small-sample overfitting and Firth-penalized estimates (Supplementary Table 3) and anterior-circulation-only sensitivity (Supplementary Table 4) should be consulted alongside this table. The ASPECTS coefficient is retained for transparency but should be interpreted cautiously because conventional ASPECTS is primarily an anterior-circulation score. Bold indicates p < 0.05.

### Procedural and safety outcomes

Procedural and safety outcomes for the full cohort are summarized in Table 3. Successful reperfusion (TICI 2b−3) was achieved in 211 of 212 patients (99.5%), with complete reperfusion (TICI 3) in 144 (67.9%). The median number of device passes was 1 (IQR 1–2). Median door-to-puncture time was 98.5 min (IQR 83.0–120.0), median puncture-to-recanalization time was 48.0 min (IQR 29.0–87.2), and median onset-to-recanalization time was 232.5 min (IQR 160.8–548.0). Any intracranial hemorrhage was documented in 29 patients (13.7%) (31), and in-hospital death occurred in 15 (7.1%).

**Table 3:** Procedural performance, safety outcomes, and short-term clinical outcomes (full cohort, N = 212).

Variable	Overall (*N* = 212)
Door-to-puncture time, min, median (IQR)	98.5 (83.0–120.0)
Puncture-to-recanalization time, min, median (IQR)	48.0 (29.0–87.2)
Onset-to-recanalization time, min, median (IQR)	232.5 (160.8–548.0)
Number of thrombectomy passes, median (IQR)	1.0 (1.0–2.0)
Stent retriever, *n* (%)	110 (51.9)
Aspiration alone, *n* (%)	18 (8.5)
Balloon angioplasty, *n* (%)	8 (3.8)
Combined techniques, *n* (%)	76 (35.8)
TICI 0–2a, *n* (%)	1 (0.5)
TICI 2b, *n* (%)	66 (31.1)
TICI 2c, *n* (%)	1 (0.5)
TICI 3, *n* (%)	144 (67.9)
Successful reperfusion (TICI 2b−3), *n* (%)	211 (99.5)
Any intracranial hemorrhage, *n* (%)	29 (13.7)
In-hospital death, *n* (%)	15 (7.1)
NIHSS score at 24 h, median (IQR)	9.0 (4.0–18.0)
Change in NIHSS, baseline to 24 h, median (IQR)	5.0 (0.0–10.8)
Length of hospital stay, days, median (IQR)	14.0 (10.0–21.0)
90-day NIHSS score, median (IQR)	3.0 (1.0–9.0)
90-day mRS score, median (IQR), *n* = 206	2.0 (1.0–4.0)
Functional independence at 90 days (mRS 0–2), *n* = 206	111 (53.9)
Sensitivity range for functional independence (best/worst/MICE-pooled, *n* = 212)	52.4%−55.2% (MICE 53.3%)

Procedural and safety outcomes apply to the full cohort (N = 212); the six patients without 90-day follow-up are included for procedural denominators. Sensitivity analyses for the missing 90-day mRS are tabulated in Supplementary Table 6.

### Exploratory regression analysis and model discrimination

In univariable analysis, baseline NIHSS score (OR 0.96, 95% CI 0.92–0.99; *p* = 0.021) and intravenous thrombolysis before thrombectomy (OR 1.99, 95% CI 1.08–3.68; *p* = 0.027) were associated with 90-day functional independence. These results are reported as observational associations.

In Model 1 (*n* = 204; events = 110), baseline NIHSS score showed an adjusted association with lower odds of functional independence (aOR 0.95, 95% CI 0.91–0.99; *p* = 0.008). Intravenous thrombolysis before thrombectomy showed a numerical adjusted association with functional independence (aOR 2.14, 95% CI 1.11–4.13; *p* = 0.024). Other baseline variables, including age, ASPECTS, vascular risk factors, and vessel territory, did not show statistically significant adjusted associations.

In Model 2 (*n* = 165; events = 86), baseline NIHSS remained associated with outcome (aOR 0.93, 95% CI 0.89–0.98; *p* = 0.004), and IVT again showed a numerical adjusted association (aOR 2.47, 95% CI 1.17–5.21; *p* = 0.018). Door-to-puncture time, puncture-to-recanalization time, and number of device passes were not statistically significant after adjustment. Because Model 2 had a smaller complete-case sample, these estimates should be considered exploratory.

#### Firth-penalized robustness

Firth-corrected estimates were directionally concordant with maximum-likelihood results for baseline NIHSS (Model 1 Firth aOR 0.95, 95% CI 0.91–0.99; Model 2 Firth aOR 0.94, 95% CI 0.89–0.98) and showed modest attenuation for IVT (Model 1 Firth aOR 2.04, 95% CI 1.09–3.91, −4.4% vs. maximum-likelihood estimation (MLE); Model 2 Firth aOR 2.29, 95% CI 1.14–4.72, −7.1% vs. MLE), confirming that the maximum-likelihood estimates were not appreciably biased by the low events-per-variable ratio (Supplementary Table 3).

#### Anterior-circulation-only sensitivity

In Model 1 restricted to anterior-circulation cases (ICA, MCA, tandem-ICA+MCA; *n* = 171; events = 90), the adjusted association of baseline NIHSS was preserved (aOR 0.95, 95% CI 0.91–0.99), the ASPECTS coefficient remained statistically non-significant (aOR 1.09, 95% CI 0.89–1.35; *p* = 0.40), and the IVT association no longer reached statistical significance (aOR 2.02, 95% CI 0.98–4.17; Firth aOR 1.94, 95% CI 0.98–3.97). The attenuation and loss of significance for IVT in the anterior-circulation subset argues for an interpretation in which the full-cohort IVT estimate reflects between-territory case-mix and confounding by indication rather than a uniform biological effect (Supplementary Table 4).

#### Intravenous thrombolysis robustness

The E-value for the Model 1 IVT point estimate (adjusted relative risk approximately 1.38) was 2.10, with an E-value of 1.29 for the lower 95% confidence bound. An unmeasured confounder linked to both IVT receipt and functional outcome by a risk ratio of at least 2.10 (point) or 1.29 (lower bound) would therefore be needed to fully explain the observed association after adjustment for the variables in Model 1 (Supplementary Table 5). Standard inverse-probability-of-treatment weighting using a propensity model for IVT receipt yielded a weighted IVT aOR of 2.33 (95% CI 1.40–3.86), but standardized mean differences (Supplementary Table 5b) showed that IPTW failed to balance several baseline covariates—onset-to-door time in particular (|SMD| = 0.74, above the 0.10 threshold) and ASPECTS (|SMD| = 0.18). The IPTW estimate therefore does not represent a balanced marginal effect and is reported only for transparency. Overlap weighting, which achieves exact balance on all propensity-score variables by construction (all |SMD| < 0.001 in our data), yielded an attenuated IVT aOR of 1.88 (95% CI 0.58–6.09) that is no longer statistically significant and that sits much closer to the pooled randomized-trial effect size (11, 12). Three independent sensitivity analyses (Firth attenuation, anterior-circulation-only restriction, and overlap weighting) all move the IVT estimate toward the null and across the conventional significance threshold—a convergence we read as supporting a confounded rather than causal interpretation of the unweighted association.

#### Discrimination and calibration

**Figure 2:**
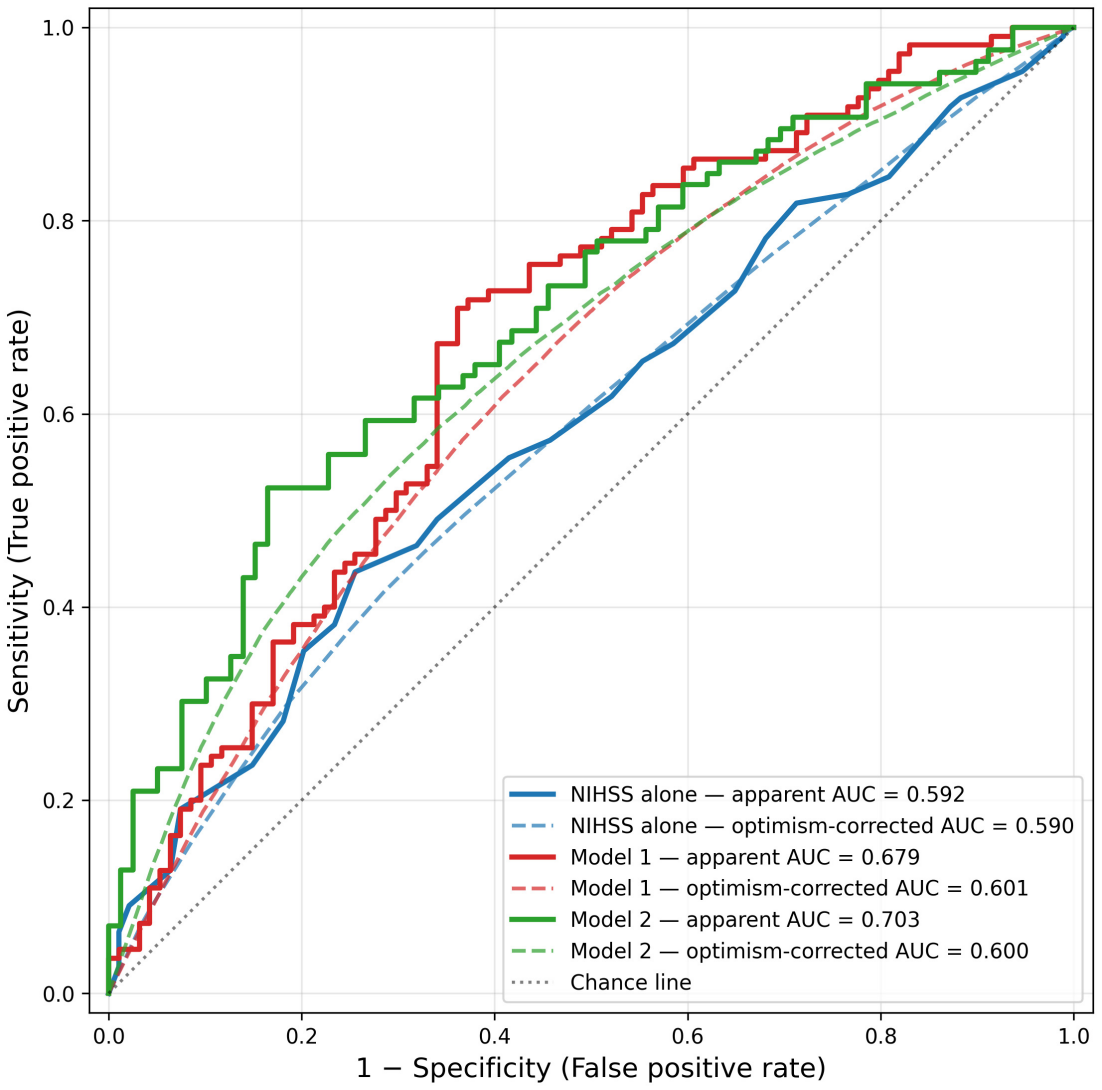
Receiver operating characteristic curves for 90-day functional independence. Curves are shown for baseline NIHSS alone (apparent C-statistic 0.592, optimism-corrected 0.590), Model 1 (apparent C-statistic 0.679, optimism-corrected 0.601), and Model 2 (apparent C-statistic 0.703, optimism-corrected 0.600). Optimism was estimated using 2,000-iteration bootstrap resampling (Harrell-style correction). Bootstrap-corrected calibration slopes were 0.56 for Model 1 and 0.47 for Model 2 (apparent slope = 1.00 by construction), and bootstrap-corrected Brier scores were 0.24 for both multivariable models, indicating that recalibration shrinkage and minimal incremental information gain over an uninformative reference are both characteristic of overfitting in low-EPV settings (see Supplementary Figure 1 for calibration curves and Supplementary Table 7 for full metrics). 95% confidence intervals for the apparent AUC were derived using bootstrap percentile resampling. The numerically higher apparent C-statistic for Model 2 should be interpreted cautiously because of the smaller complete-case sample (*n* = 165, events = 86) and the higher optimism inflation in low-EPV settings. The curves describe apparent and internally validated discrimination within this single-center cohort only and do not represent external validation of a clinical prediction model. Receiver operating characteristic curves for a 90-day functional independence: apparent (solid) and 2,000-iteration bootstrap-corrected (dashed) discrimination. NIHSS, National Institutes of Health Stroke Scale; AUC, area under the curve.

The NIHSS-alone reference model yielded an apparent C-statistic of 0.592 (optimism-corrected 0.590); Model 1 had an apparent C-statistic of 0.679 (optimism-corrected 0.601); and Model 2 had an apparent C-statistic of 0.703 (optimism-corrected 0.600). The optimism gaps (0.08 for Model 1 and 0.10 for Model 2) confirm that the apparent discrimination of the multivariable models reflects in-sample overfitting expected at low events-per-variable; corrected discrimination converges to a modest C-statistic near 0.60 once optimism is removed (Supplementary Figure 1; Supplementary Table 7). Bootstrap-corrected calibration slopes were 0.56 for Model 1 and 0.47 for Model 2 (vs. a mathematically apparent slope of 1.00), pointing to severe shrinkage of the linear predictor when applied beyond the training sample—a pattern typical of overfitting in low-EPV regression. Bootstrap-corrected Brier scores were 0.24 for both multivariable models, only marginally better than a non-informative reference of 0.25 (Supplementary Table 7). Figure 2 shows the corresponding receiver operating characteristic (ROC) curves. We do not interpret either model as a deployable clinical prediction tool.

#### Collateral circulation sensitivity (*post-hoc*)

When the pre-procedural Higashida 0–4 CTA collateral score (27) was added to Model 1 (*n* = 203, events = 110), the collateral coefficient was not statistically significant (aOR 0.92, 95% CI 0.66–1.30; *p* = 0.65) and the NIHSS coefficient was unchanged (aOR 0.95). The IVT coefficient remained directionally similar (aOR 2.25, 95% CI 1.16–4.39; Firth aOR 2.14, 95% CI 1.13–4.12), so the observed IVT association is not explained by between-group collateral status. The null collateral coefficient mirrors the narrow distribution of the collateral scale in this cohort (median 2, IQR 1–3) and the broader observation that collateral grading is a coarse imaging surrogate (Supplementary Table 8) (34).

#### Prior stroke proxy for pre-stroke functional status (*post-hoc*)

Because pre-stroke modified Rankin Scale was not systematically recorded, we added documented history of prior stroke or transient ischemic attack (12.3% prevalence in the analytic cohort) as a sensitivity covariate in Model 1 (*n* = 204, events = 110). The prior-stroke coefficient was not statistically significant (aOR 0.97, 95% CI 0.39–2.41; *p* = 0.94), and the baseline NIHSS and IVT coefficients were unchanged (NIHSS aOR 0.95, IVT aOR 2.14; Supplementary Table 10). The unchanged primary coefficients suggest that, within the limits of this binary proxy, residual confounding by gross pre-stroke functional impairment is unlikely to explain the present results. Prior-stroke history does not, however, capture the full spectrum of pre-stroke disability, and a structured pre-stroke mRS would clearly be preferable.

## Discussion

**Figure 3:**
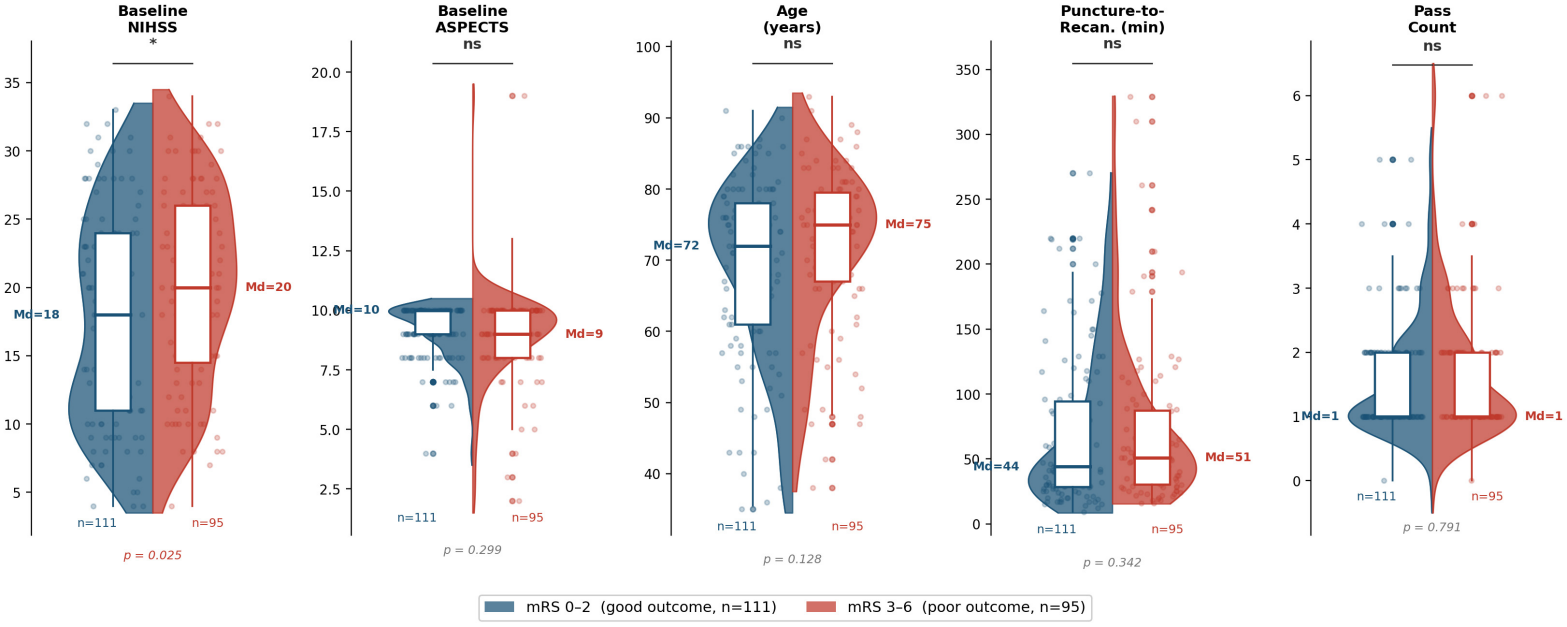
Distribution of key clinical and procedural variables stratified by 90-day functional outcome (Primary Analysis Cohort, *N* = 206). Between-group comparisons used the Mann-Whitney *U*-test. These distributions provide descriptive context for the registry findings. Baseline NIHSS was the only displayed variable with a statistically significant between-group difference. ASPECTS results are interpreted cautiously because posterior-circulation cases were included and pc-ASPECTS was not systematically recorded. mRS, modified Rankin Scale; NIHSS, National Institutes of Health Stroke Scale; ASPECTS, alberta stroke program early CT score. *P < 0.05.

In this retrospective single-center registry of 212 patients undergoing mechanical thrombectomy for acute LVO stroke, 53.9% of patients with available 90-day follow-up achieved functional independence, and 99.5% achieved successful reperfusion. Distributions of key clinical and procedural variables stratified by 90-day functional outcome are shown in Figure 3. Across alternative handling of missing follow-up the functional-independence proportion ranged from 52.4% to 55.2%, with multiple imputation yielding 53.3%. Baseline NIHSS was the most consistent clinical correlate of outcome and held up across full-cohort, anterior-circulation-only, Firth-penalized, and collateral-adjusted analyses. Intravenous thrombolysis before thrombectomy showed an adjusted association with functional independence in the full cohort, but lost statistical significance in the anterior-circulation subset and is best read as a confounded observational finding.

The relationship between baseline NIHSS score and functional outcome after thrombectomy is clinically plausible and agrees with Highly Effective Reperfusion Evaluated in Multiple Endovascular Stroke Trials (HERMES) collaboration analyses (24), German Stroke Registry data (25), and a recent systematic review of EVT prediction models (35). NIHSS summarizes neurological deficit severity at presentation and may reflect—imperfectly—ischemic burden, collateral status, and neurological reserve. The modest discrimination of the NIHSS-alone reference model in this cohort (apparent and optimism-corrected C-statistic ≈ 0.59) echoes prior reports: baseline NIHSS is a reliable group-level correlate of outcome but an inadequate standalone individual-level prognostic tool (35).

Age and ASPECTS did not show statistically significant adjusted associations with functional independence in either Model 1 or its anterior-circulation-only counterpart. This does not mean that age or imaging burden are unimportant. The ASPECTS distribution in our cohort was narrow, which limited discrimination. ASPECTS is also a coarse measure and does not capture infarct location, collateral status, perfusion mismatch, or tissue eloquence. Recent secondary analyses from the Endovascular Therapy in Acute Anterior Circulation Large Vessel Occlusive Patients with a Large Infarct Core (ANGEL-ASPECT) (36) and Efficacy and Safety of Thrombectomy in Stroke With Extended Lesion and Extended Time Window (TENSION) (34) trials suggest that ASPECTS in the low range (2–5) no longer reliably stratifies thrombectomy benefit, and that infarct volume and postacute neurological status emerge as relevant beyond conventional ASPECTS scoring.

Our anterior-circulation-only sensitivity analysis (aOR 1.09, 95% CI 0.89–1.35) shows that the null ASPECTS coefficient was not driven by inclusion of posterior-circulation cases—it holds within the anatomical range for which ASPECTS was developed. A *post-hoc* Model 1 that added the pre-procedural Higashida CTA collateral score (27) showed no independent collateral effect and left the NIHSS and IVT coefficients unchanged, arguing against residual confounding by gross collateral status. Together these observations weaken the case for using a single ASPECTS threshold as a procedural gatekeeper (37) and argue for multimodal selection in routine practice.

The observed association between intravenous thrombolysis before thrombectomy and functional independence calls for careful interpretation. Patients who receive thrombolysis differ in important ways from those who do not. They may arrive earlier through coordinated pre-hospital triage, have fewer contraindications, be less likely to be anticoagulated, or follow different referral pathways. The registry did not fully capture these factors.

Our unweighted adjusted odds ratio (2.14 in Model 1, 2.47 in Model 2) is much larger than the effect sizes reported in randomized non-inferiority trials of EVT alone vs. bridging IVT [Direct Mechanical Thrombectomy in Acute LVO Stroke (SKIP) (9) and related trials] and in subsequent individual-patient-data meta-analyses (11, 12). Those pooled randomized data support a small and time-dependent benefit of bridging alteplase—relative risk approximately 1.05–1.15, significant only when alteplase is delivered within about 140 min of onset. The gap between our registry estimate and the pooled randomized estimate is, in our reading, the expected fingerprint of confounding by indication.

Four internal observations reinforce that interpretation. First, Firth-penalized estimation attenuated the IVT coefficient by 4%−7%, suggesting some small-sample bias inflation. Second, restriction to anterior-circulation cases dropped the IVT estimate below conventional statistical significance (aOR 2.02, 95% CI 0.98–4.17); this loss of significance reflects both a reduced sample (*n* down 16%, events down 18%) and territory-dependent case mix, and we cannot disentangle the two contributions in this dataset. Third, although standard IPTW did not attenuate the estimate, the IPTW model itself failed to balance several baseline covariates—onset-to-door time most of all, |SMD| = 0.74—so an apparently unchanged IPTW estimate under such imbalance carries little weight as evidence against unmeasured confounding. Fourth, and most decisively, overlap weighting (which by construction achieves exact balance on all propensity-score covariates) attenuated the IVT estimate to aOR 1.88 (95% CI 0.58–6.09), removed statistical significance, and pulled the estimate close to the pooled randomized-trial effect size. The E-value of 2.10 for the unweighted point estimate (1.29 for the lower confidence bound) further quantifies how much additional unmeasured confounding could explain any residual association (8); this magnitude is within the range plausibly produced by pre-hospital triage protocols, anticoagulation history, prior intracranial hemorrhage, and contraindication-based selection.

Two contemporary tenecteplase trials inform the evolving bridging evidence base and help clarify the road ahead. The Thrombectomy With Versus Without recombinant human tenecteplase tissue-type plasminogen activator in Stroke (BRIDGE-TNK) trial (38) randomized patients to intravenous tenecteplase vs. standard care before thrombectomy and showed improved functional outcomes with bridging tenecteplase. The Tenecteplase Reperfusion Therapy in Acute Ischemic Cerebrovascular Events III (TRACE III) trial (39) tested intravenous tenecteplase for ischemic stroke at 4.5–24 h without thrombectomy and showed benefit relative to standard care. Taken together, these trials extend the bridging and thrombolysis evidence base beyond alteplase and suggest that local registries collecting structured tenecteplase exposure data may soon contribute to important practice questions. Until such randomized evidence converges, treatment decisions for individual patients should continue to follow randomized evidence and local protocols rather than the present observational point estimate.

Workflow metrics did not show statistically significant adjusted associations with functional independence after baseline adjustment. This null should not be read as evidence that workflow is unimportant. The median door-to-puncture time of 98.5 min exceeds The Joint Commission CSTK-09 (Comprehensive Stroke measure 09: Arrival Time to Skin Puncture) institutional benchmark for skin puncture (40) and is longer than the per-trial medians reported in pooled HERMES workflow analyses (28), flagging a concrete local quality-improvement target. The high reperfusion rate, limited sample size, and restricted variation in procedural times together reduced our analytic power to detect a time–outcome relationship in this cohort. Continued reduction of door-to-imaging, door-to-puncture, and skin-puncture-to-reperfusion intervals remains a quality-improvement priority for EVT programs, similar in principle to door-to-needle time for IVT (16).

### Reperfusion–outcome dissociation: an unexpected internal benchmark

A salient and previously under-discussed feature of this single-center cohort is the dissociation between procedural success and functional recovery. Despite a TICI 2b−3 rate of 99.5%—well above HERMES (71%) (1, 41), Systematic Evaluation of Patients Treated With Neurothrombectomy Devices for Acute Ischemic Stroke (STRATIS) (88%) (23), Multicenter Randomized Clinical Trial of Endovascular Treatment for Acute Ischemic Stroke in the Netherlands (MR CLEAN) Registry (84–87%), and Endovascular Treatment Key Technique and Emergency Work Flow Improvement of Acute Ischemic Stroke (ANGEL-ACT) (89%)–45.9% of reperfused patients with available 90-day follow-up failed to achieve functional independence. This pattern parallels the increasingly recognized phenomenon of clinically ineffective reperfusion, in which restoration of macrovascular patency does not translate into tissue-level reperfusion or clinical recovery, owing to microvascular obstruction, capillary stalls, distal embolization, or completed infarction in eloquent territory (14, 15).

The exceptionally high TICI 2b−3 rate in this cohort should be read with caution. Several contributors deserve explicit acknowledgment. First, this high-volume center may have practiced selective case acceptance, declining or transferring out technically challenging cases (very-distal occlusions, severe atherosclerotic tandem disease, or anatomically inaccessible vessels) that lower the recanalization rate in larger multicenter registries. Second, TICI grading was assigned retrospectively from procedural records by the treating team rather than by an independent adjudication committee, which can inflate measured success rates relative to studies with central core-lab adjudication (1). Third, the small cohort size concentrates operator and case-selection effects to a degree that limits direct comparability with multicenter registries. These caveats do not erase the observed reperfusion–outcome gap—which is internally informative regardless of selection—but they place the headline 99.5% figure in appropriate context. The present descriptive data extend the futile-reperfusion literature with a quantitative single-center reperfusion–outcome gap. They cannot, however, evaluate a no-reflow mechanism, which would require perfusion imaging or transcranial Doppler data that the registry does not contain.

### Practical contribution

This study provides a local real-world benchmark for EVT practice in a regional Chinese stroke center. Its value lies in (i) documenting outcome rates, reperfusion performance, safety events, and workflow metrics in routine care; (ii) showing a quantitative reperfusion–outcome gap in a near-universal-recanalization setting that informs the broader futile-reperfusion literature; and (iii) offering a worked example of how exploratory associations from a low-EPV registry should be presented (Firth penalization, sensitivity restriction, E-value, IPTW, MICE for missing outcomes, optimism correction) rather than reported as a prediction model. The results are most useful for institutional review and comparison with broader EVT datasets, not for proposing a new prognostic score or proving a treatment effect.

## Limitations

Several limitations should be kept in mind. First, the retrospective single-center design precludes causal inference and limits generalizability to centers with different patient populations, transfer patterns, imaging protocols, and operator experience. Second, the regression models were constrained by the number of events relative to the number of parameters. We addressed this with Firth penalized estimation (Supplementary Table 3), which gave directionally concordant but slightly attenuated estimates, and we explicitly do not interpret either model as a deployable prediction tool. Third, pre-stroke functional status was not systematically recorded, which may have affected both treatment selection and 90-day outcome. Fourth, six patients lacked 90-day mRS and had worse baseline profiles; we addressed this by reporting four scenarios (best-case 55.2%, complete-case 53.9%, worst-case 52.4%, MICE-pooled 53.3%, Supplementary Table 6), all of which converge within a narrow band.

Fifth, conventional ASPECTS is primarily an anterior-circulation score, whereas the cohort included posterior-circulation cases and pc-ASPECTS was not systematically available; the anterior-circulation-only sensitivity analysis confirmed that the non-significant ASPECTS coefficient and the preserved NIHSS coefficient were not artifacts of cross-territory mixing. Sixth, the thrombolysis association is subject to confounding by indication and to unmeasured factors such as onset-to-treatment time, contraindications, anticoagulation status, previous hemorrhage, and referral pathway. We addressed this with E-value computation, two propensity-score adjustments (IPTW and overlap weighting), and an anterior-circulation-only sensitivity analysis; standard IPTW failed to balance baseline covariates (onset-to-door time most of all, |SMD| = 0.74) and is therefore reported only for transparency, whereas overlap weighting achieved exact balance and attenuated the IVT association to non-significance (aOR 1.88, 95% CI 0.58–6.09). Even after these adjustments, residual unmeasured confounding remains possible. Seventh, intracranial hemorrhage could not be separated into symptomatic and asymptomatic events. Eighth, occlusion-site sub-segmentation (M1 vs. M2 within MCA, ICA segment, basilar segment) was available in the source digital subtraction angiography reports but was not systematically coded in the registry export and could not be retrieved within the timeframe of this revision; this limits direct comparison with HERMES sub-analyses of medium-vessel occlusion (41). Ninth, the registry did not contain structured Trial of Org 10172 in Acute Stroke Treatment (TOAST) etiologic classification, discharge NIHSS, anesthetic management, carotid stenosis grading, or systematic post-acute care indicators, which would have supported deeper comparability with international registries; prospective collection of these fields is a clear priority. Tenth, in the absence of perfusion imaging or transcranial Doppler, the reperfusion–outcome dissociation reported here is descriptive only and should not be read as direct evidence of the no-reflow phenomenon.

## Conclusion

In this single-center retrospective registry of patients undergoing mechanical thrombectomy for acute large-vessel occlusion stroke, baseline NIHSS was the most consistent clinical correlate of 90-day functional independence and held up across Firth-penalized estimation, anterior-circulation-only restriction, and collateral-adjusted models. Intravenous thrombolysis before thrombectomy showed an adjusted association with functional independence in the unweighted analysis (aOR 2.14, 95% CI 1.11–4.13) that attenuated to non-significance under overlap weighting once exact covariate balance was achieved (aOR 1.88, 95% CI 0.58–6.09), pulling the present registry estimate close to the pooled randomized-trial relative risk of 1.05–1.15. Together with the E-value of 2.10 and the loss of significance in the anterior-circulation subset, these analyses point to confounding by indication as the most plausible explanation for the unweighted IVT association. The 99.5% TICI 2b−3 reperfusion rate, paired with the observation that 45.9% of reperfused patients did not regain functional independence, situates this cohort within the broader contemporary discussion of clinically ineffective reperfusion. We view the study as a local benchmark for EVT outcomes, safety, and workflow that supports continued quality review of door-to-EVT processes (16) and prospective evaluation of futile-reperfusion mechanisms—with the limits of retrospective single-center data clearly in mind.

## Data Availability

The raw data supporting the conclusions of this article will be made available by the authors, without undue reservation.
