# Supplementary material for: Clinical correlates, procedural benchmarks, and reperfusion–outcome dissociation after mechanical thrombectomy for acute large-vessel occlusion stroke: a single-center retrospective registry study
Source: Front Neurol. 2026 Jul 30;17:1835315. doi: 10.3389/fneur.2026.1835315 (PMC13467987; doi:10.3389/fneur.2026.1835315)
Supplement: Supplementary file 1 [file Supplementary_file_1.docx]

# Supplementary Materials

## Supplementary Table 1. Comparison of patients with and without available 90-day mRS data

| Variable | mRS available (n=206) | mRS missing (n=6) | P value |
| --- | --- | --- | --- |
| Age, years, mean ± SD | 70.5 ± 12.1 | 79.7 ± 7.7 | 0.032* |
| Male sex, n (%) | 124 (60.2) | 2 (33.3) | 0.226 |
| Diabetes mellitus, n (%) | 50 (24.4) | 1 (16.7) | 1.000 |
| Hypertension, n (%) | 134 (65.0) | 2 (33.3) | 0.191 |
| Atrial fibrillation, n (%) | 77 (37.4) | 3 (50.0) | 0.675 |
| Baseline NIHSS, median (IQR) | 19.0 (12.0–25.0) | 25.5 (21.8–27.8) | 0.037* |
| Baseline ASPECTS, median (IQR) | 9.0 (9.0–10.0) | 10.0 (9.2–10.0) | 0.375 |
| IV thrombolysis before thrombectomy, n (%) | 64 (31.2) | 2 (33.3) | 1.000 |
| Door-to-puncture time, min, median (IQR) | 98.5 (83.0–120.0) | 111.5 (90.8–149.5) | 0.310 |
| Puncture-to-recanalization time, min, median (IQR) | 47.0 (29.0–88.8) | 73.0 (55.2–81.0) | 0.233 |
| Successful reperfusion (TICI 2b–3), n (%) | 205 (99.5) | 6 (100.0) | 1.000 |
| Any intracranial hemorrhage, n (%) | 27 (13.1) | 2 (33.3) | 0.191 |
| In-hospital death, n (%) | 12 (5.8) | 3 (50.0) | 0.005* |

** p < 0.05. Significant differences in baseline NIHSS and in-hospital mortality indicate that missing 90-day mRS data were unlikely to be missing completely at random. These findings support the conservative missing-outcome scenario analyses presented in Supplementary Table 6.*

## Supplementary Table 3. Firth penalized maximum-likelihood estimation vs standard maximum-likelihood estimates (Model 1 and Model 2)

| Variable | Model 1 MLE (95% CI) | Model 1 Firth (95% CI) | Δ% | Model 2 MLE (95% CI) | Model 2 Firth (95% CI) | Δ% |
| --- | --- | --- | --- | --- | --- | --- |
| Age (per year) | 0.99 (0.96–1.02) | 0.99 (0.97–1.02) | +0.1% | 0.99 (0.96–1.02) | 0.99 (0.96–1.02) | +0.1% |
| Male sex | 0.95 (0.51–1.77) | 0.96 (0.52–1.74) | +0.4% | 0.89 (0.43–1.82) | 0.90 (0.45–1.77) | +1.2% |
| Baseline NIHSS (per point) | 0.95 (0.91–0.99) | 0.95 (0.91–0.99) | +0.4% | 0.93 (0.89–0.98) | 0.94 (0.89–0.98) | +0.7% |
| Baseline ASPECTS (per point) | 1.09 (0.90–1.33) | 1.08 (0.90–1.32) | -1.0% | 1.11 (0.90–1.37) | 1.09 (0.90–1.34) | -1.5% |
| Atrial fibrillation | 0.87 (0.46–1.67) | 0.88 (0.47–1.65) | +0.4% | 0.80 (0.37–1.70) | 0.82 (0.40–1.68) | +2.5% |
| Diabetes mellitus | 1.65 (0.81–3.36) | 1.58 (0.80–3.17) | -4.3% | 1.16 (0.51–2.64) | 1.13 (0.52–2.48) | -2.9% |
| Hypertension | 0.58 (0.30–1.10) | 0.60 (0.32–1.11) | +3.8% | 0.52 (0.25–1.08) | 0.55 (0.27–1.10) | +6.5% |
| IV thrombolysis before EVT | 2.14 (1.11–4.13) | 2.04 (1.09–3.91) | -4.4% | 2.47 (1.17–5.21) | 2.29 (1.14–4.72) | -7.1% |
| ICA vs MCA | 1.39 (0.70–2.78) | 1.36 (0.70–2.68) | -2.2% | 0.93 (0.42–2.04) | 0.94 (0.44–1.99) | +0.7% |
| Tandem ICA+MCA vs MCA | 1.04 (0.27–4.06) | 1.04 (0.28–3.87) | -0.2% | 0.79 (0.17–3.59) | 0.82 (0.19–3.34) | +3.6% |
| Basilar vs MCA | 1.46 (0.53–4.00) | 1.41 (0.54–3.80) | -3.5% | 1.02 (0.33–3.19) | 1.01 (0.34–3.03) | -0.5% |
| Other vs MCA | 2.11 (0.49–9.00) | 1.89 (0.52–8.35) | -10.3% | 2.22 (0.33–14.77) | 1.88 (0.37–12.60) | -15.1% |
| Door-to-puncture time (per min) | — | — | — | 0.99 (0.99–1.00) | 1.00 (0.99–1.00) | +0.1% |
| Puncture-to-recan time (per min) | — | — | — | 1.00 (0.99–1.00) | 1.00 (0.99–1.00) | +0.0% |
| Number of thrombectomy passes | — | — | — | 1.00 (0.66–1.50) | 0.99 (0.68–1.47) | -0.2% |

*Firth penalized estimation reduces small-sample bias in logistic regression with low events-per-variable (Heinze & Schemper 2002). 95% confidence intervals for Firth estimates are profile-penalized-likelihood intervals. Directional concordance and modest attenuation (≤7%) of point estimates support that the conventional MLE estimates were not appreciably biased by EPV < 10 in either model. NIHSS and ASPECTS coefficients were unchanged across methods; the IVT coefficient was attenuated by 4.4% in Model 1 and 7.1% in Model 2.*

## Supplementary Table 4. Anterior-circulation-only sensitivity Model 1 (n = 171; events = 90)

| Variable | Full Model 1 MLE aOR (95% CI) | Anterior-only MLE aOR (95% CI) | Anterior-only Firth aOR (95% CI) |
| --- | --- | --- | --- |
| Age (per year) | 0.99 (0.96–1.02) | 1.00 (0.97–1.03) | 1.00 (0.97–1.03) |
| Male sex | 0.95 (0.51–1.77) | 0.99 (0.51–1.92) | 0.99 (0.52–1.88) |
| Baseline NIHSS (per point) | 0.95 (0.91–0.99) | 0.95 (0.91–0.99) | 0.95 (0.91–0.99) |
| Baseline ASPECTS (per point) | 1.09 (0.90–1.33) | 1.09 (0.89–1.35) | 1.08 (0.89–1.34) |
| Atrial fibrillation | 0.87 (0.46–1.67) | 0.80 (0.40–1.58) | 0.81 (0.42–1.57) |
| Diabetes mellitus | 1.65 (0.81–3.36) | 1.59 (0.73–3.47) | 1.53 (0.73–3.28) |
| Hypertension | 0.58 (0.30–1.10) | 0.52 (0.25–1.08) | 0.54 (0.27–1.09) |
| IV thrombolysis before EVT | 2.14 (1.11–4.13) | 2.02 (0.98–4.17) | 1.94 (0.98–3.97) |
| ICA vs MCA | 1.39 (0.70–2.78) | 1.39 (0.70–2.79) | 1.36 (0.70–2.69) |
| Tandem ICA+MCA vs MCA | 1.04 (0.27–4.06) | 1.04 (0.27–4.05) | 1.04 (0.28–3.86) |

*Anterior-circulation-only Model 1 restricts the analysis to ICA, MCA, and tandem-ICA+MCA cases, excluding 35 basilar/other-territory patients (16% of the cohort). The Basilar and Other vessel dummies are dropped from this model. Key findings: baseline NIHSS coefficient is preserved (aOR 0.95, 95% CI 0.91–0.99); ASPECTS remains non-significant (aOR 1.09, 95% CI 0.89–1.35; p = 0.40), confirming that the null ASPECTS finding in the full cohort is not driven by cross-territory mixing; IVT coefficient is attenuated and no longer statistically significant (aOR 2.02, 95% CI 0.98–4.17), indicating that the full-cohort IVT signal is partly driven by between-territory case-mix.*

## Supplementary Table 5. Intravenous thrombolysis robustness: E-value, IPTW, and overlap weighting

| Analysis | aOR (95% CI) | Approximate aRR | E-value (point) | E-value (lower CI bound) |
| --- | --- | --- | --- | --- |
| Model 1 MLE (unweighted) | 2.14 (1.11–4.13) | 1.38 | 2.10 | 1.29 |
| Model 2 MLE (unweighted) | 2.47 (1.17–5.21) | 1.44 | 2.24 | 1.37 |
| **Model 1 covariates, standard IPTW (truncated 1%/99%)** | **2.33 (1.40–3.86)** | — | — | — |
| **Model 1 covariates, overlap weighting** | **1.88 (0.58–6.09)** | — | — | — |

*E-value calculated per VanderWeele & Ding (2017) after converting the adjusted OR to an approximate adjusted RR using the baseline outcome rate in the unexposed group (0.486 in this cohort). The Model 1 E-value of 2.10 indicates that an unmeasured confounder associated with both IVT receipt and functional outcome by a risk ratio of at least 2.10 would be required to fully explain the observed point association; the lower-CI E-value of 1.29 indicates that a much weaker unmeasured confounder is enough to remove statistical significance. Two complementary propensity-score (PS) adjustments are reported using a PS model with age, sex, baseline NIHSS, ASPECTS, AF, DM, HTN, and onset-to-door time: (i) standard IPTW with weights truncated at the 1st/99th percentile, and (ii) overlap weighting (Li, Morgan & Zaslavsky 2018, ref 41). Standardized mean differences before and after weighting are reported in Supplementary Table 5b; standard IPTW failed to balance several covariates (onset-to-door time in particular, |SMD| = 0.74) and the IPTW estimate is therefore not a balanced marginal effect and is reported only for transparency. Overlap weighting achieved exact balance on all PS covariates (all |SMD| < 0.001 by construction) and attenuated the IVT aOR to 1.88 (95% CI 0.58–6.09) — no longer statistically significant, and close to the pooled randomized-trial effect size (RR ≈ 1.05–1.15) [31,32]. Three independent sensitivity analyses (Firth attenuation, anterior-circulation-only restriction, and overlap weighting) all move the IVT estimate toward the null, supporting a confounded rather than causal interpretation of the unweighted association.*

## Supplementary Table 5b. Covariate balance (standardized mean differences) before and after propensity-score weighting

| Covariate | Unweighted | SMD |  |
| --- | --- | --- | --- |
| Age | 0.255 | 0.009 | <0.001 |
| Male sex | 0.100 | 0.045 | <0.001 |
| Baseline NIHSS | 0.108 | 0.016 | <0.001 |
| Baseline ASPECTS | 0.317 | **0.182** | <0.001 |
| Atrial fibrillation | 0.067 | **0.113** | <0.001 |
| Diabetes mellitus | 0.161 | 0.002 | <0.001 |
| Hypertension | 0.116 | 0.059 | <0.001 |
| Onset-to-door time | 1.088 | **0.741** | <0.001 |
| **Maximum | SMD | ** | **1.088** |

*Standardized mean differences computed per Yang & Dalton (2012). Threshold of acceptable balance: |SMD| < 0.10 (bold values exceed this threshold). Standard IPTW failed to balance ASPECTS (|SMD| = 0.182), atrial fibrillation (|SMD| = 0.113), and especially onset-to-door time (|SMD| = 0.741); the IPTW-weighted IVT estimate is therefore not a valid marginal effect under this weighting scheme. Overlap weighting achieves exact balance on all eight propensity-score covariates by construction (residual |SMD| reflects floating-point arithmetic only) and is the preferred sensitivity analysis for the IVT association in this cohort.*

## Supplementary Table 6. Missing 90-day mRS — four-scenario sensitivity analysis

| Scenario | Functional independence (n) | Denominator | Functional independence rate |
| --- | --- | --- | --- |
| Complete-case | 111 | 206 | 53.9% |
| Best-case (missing = independent) | 117 | 212 | 55.2% |
| Worst-case (missing = poor) | 111 | 212 | 52.4% |
| MICE-pooled (20 imps) | 113 | 212 | 53.3% |

*Multiple imputation by chained equations used baseline NIHSS, age, ASPECTS, atrial fibrillation, IVT, reperfusion success, ICH, and in-hospital death as predictors, with 20 imputations. The range of imputed functional-independence rates across the 20 imputations was 52.4–54.2%, indicating high stability. All four scenarios converge within a 2.8-percentage-point band, supporting that the primary complete-case estimate is robust to plausible missing-outcome handling.*

## Supplementary Table 7. Apparent and optimism-corrected discrimination and calibration metrics

| Model | Apparent AUC | Optimism-corrected AUC | Apparent calibration slope | Bootstrap-corrected calibration slope | Apparent Brier | Bootstrap-corrected Brier |
| --- | --- | --- | --- | --- | --- | --- |
| NIHSS alone | 0.592 | 0.590 | 1.00 | 1.05 | 0.242 | 0.244 |
| Model 1 | 0.679 | 0.601 | 1.00 | **0.56** | 0.225 | 0.240 |
| Model 2 | 0.703 | 0.600 | 1.00 | **0.47** | 0.217 | 0.240 |

*Optimism estimated using Harrell-style 2,000-iteration bootstrap with replacement. Apparent calibration slope is mathematically 1.0 by construction when logistic regression is recalibrated against its own predicted linear predictor on the training data; this value carries no diagnostic information. The bootstrap-corrected calibration slope is the mean of the recalibration slopes obtained when bootstrap models are evaluated on the original cohort and provides a meaningful measure of overfitting; values well below 1.0 indicate that the model’s predicted linear predictor must be shrunk before use outside the training sample. Model 1 (slope 0.56) and Model 2 (slope 0.47) both show pronounced shrinkage, the expected pattern for low-EPV overfitting. Bootstrap-corrected Brier scores for both multivariable models are 0.24, only marginally better than the uninformative reference of 0.25 (for an event rate near 50%). Bootstrap iteration counts vary because a small number of bootstrap draws produced separation that prevented logistic regression convergence and were dropped; effective iteration counts were 1979 (Model 1) and 1945 (Model 2). See Supplementary Figure 1 for calibration and ROC curves.*

## Supplementary Table 8. Post-hoc Model 1 incorporating pre-procedural Higashida 0–4 CTA collateral score (n = 203; events = 110)

| Variable | MLE aOR (95% CI) | MLE p | Firth aOR (95% CI) | Firth p |
| --- | --- | --- | --- | --- |
| Age (per year) | 0.99 (0.97–1.02) | 0.549 | 0.99 (0.97–1.02) | 0.558 |
| Male sex | 0.93 (0.50–1.73) | 0.820 | 0.94 (0.51–1.71) | 0.830 |
| Baseline NIHSS (per point) | 0.95 (0.91–0.99) | 0.009 | 0.95 (0.91–0.99) | 0.010 |
| Baseline ASPECTS (per point) | 1.09 (0.89–1.32) | 0.408 | 1.08 (0.90–1.31) | 0.430 |
| Atrial fibrillation | 0.87 (0.45–1.68) | 0.675 | 0.87 (0.46–1.65) | 0.675 |
| Diabetes mellitus | 1.61 (0.79–3.28) | 0.193 | 1.54 (0.78–3.09) | 0.214 |
| Hypertension | 0.59 (0.31–1.12) | 0.106 | 0.61 (0.32–1.13) | 0.117 |
| IV thrombolysis before EVT | 2.25 (1.16–4.39) | 0.017 | 2.14 (1.13–4.12) | 0.019 |
| ICA vs MCA | 1.34 (0.61–2.90) | 0.464 | 1.31 (0.62–2.79) | 0.480 |
| Tandem ICA+MCA vs MCA | 0.92 (0.21–4.03) | 0.914 | 0.93 (0.22–3.82) | 0.911 |
| Basilar vs MCA | 1.34 (0.46–3.94) | 0.596 | 1.30 (0.46–3.73) | 0.617 |
| Other vs MCA | 2.01 (0.46–8.78) | 0.353 | 1.80 (0.48–8.06) | 0.389 |
| Collateral score (per 1-unit) | 0.92 (0.66–1.30) | 0.649 | 0.93 (0.67–1.29) | 0.659 |

*Post-hoc analysis added after re-examination of the original registry found that pre-procedural collateral circulation score (0–4 scale) was in fact available for 211 of 212 patients; this had been incorrectly listed as unavailable in V4 Limitations. Adding the collateral score to Model 1 did not change the baseline NIHSS coefficient (aOR 0.95 unchanged) and only minimally affected the IVT coefficient (MLE: 2.14 → 2.25; Firth: 2.04 → 2.14). The collateral coefficient itself was not statistically significant (aOR 0.92, 95% CI 0.66–1.30; p = 0.65), consistent with the narrow within-cohort distribution (median 2, IQR 1–3) and with literature noting collateral grading as a coarse imaging surrogate. The principal interpretation is that the primary NIHSS and IVT associations are not confounded by gross collateral status.*

## Supplementary Figure 1. Calibration and discrimination of regression models

Panel A shows apparent calibration curves (mean predicted probability vs observed proportion functionally independent, binned by quantile). Panel B shows ROC curves with apparent and optimism-corrected AUC. Both panels confirm that the multivariable models exhibit substantial overfitting (optimism 0.08–0.10) characteristic of low-EPV settings; optimism-corrected discrimination is approximately 0.60 for both Model 1 and Model 2, only marginally better than the NIHSS-alone reference model (0.59). NIHSS = National Institutes of Health Stroke Scale.

## Supplementary Table 10. Prior stroke / TIA as a proxy for pre-stroke functional status: post-hoc Model 1 sensitivity (n = 204; events = 110)

| Variable | MLE aOR (95% CI) | MLE p | Firth aOR (95% CI) | Firth p |
| --- | --- | --- | --- | --- |
| Baseline NIHSS (per point) | 0.95 (0.91–0.99) | 0.009 | 0.95 (0.91–0.99) | 0.010 |
| IV thrombolysis before EVT | 2.14 (1.11–4.13) | 0.024 | 2.04 (1.09–3.89) | 0.026 |
| Baseline ASPECTS (per point) | 1.09 (0.89–1.33) | 0.395 | 1.08 (0.90–1.32) | 0.417 |
| Prior stroke or TIA | 0.97 (0.39–2.41) | 0.940 | 0.97 (0.40–2.35) | 0.951 |

*Prior stroke or TIA history (12.3% prevalence in the analytic cohort) is the only available proxy for pre-stroke functional status in this registry. When added as a sensitivity covariate to Model 1, the prior-stroke coefficient was non-significant (aOR 0.97), and the primary NIHSS and IVT coefficients were unchanged versus the original Model 1 (NIHSS aOR 0.95 vs 0.95; IVT aOR 2.14 vs 2.14). Within the limits of this binary proxy, residual confounding by gross pre-stroke functional impairment is unlikely to explain the observed associations; however, prior-stroke history does not capture the full spectrum of pre-stroke disability, and structured pre-stroke mRS recording remains a registry gap for future prospective work.*

## Supplementary Table 9. Comparison of TICI 2b–3 reperfusion rates across published EVT cohorts

| Cohort | N | TICI 2b–3 rate (%) |
| --- | --- | --- |
| Present study (Haining, 2023-2025) | 212 | 99.5% |
| HERMES pooled (2016) | 1287 | 71.0% |
| STRATIS Registry (US, 2017) | 984 | 87.9% |
| MR CLEAN Registry (NL, 2017-2019) | 3279 | 85.0% |
| ANGEL-ACT (China, 2017-2019) | 1793 | 89.2% |

*Benchmark values for HERMES, STRATIS, MR CLEAN Registry, and ANGEL-ACT are from published reports; the reports vary in case definition and inclusion criteria. The 99.5% TICI 2b–3 rate observed in the present single-center cohort is unusually high relative to all of these reference series; possible contributors include case selection (declining or transferring out the most technically challenging cases), high operator experience concentrated on a small case volume, and bias-prone retrospective grading. The coexistence of this unusually high recanalization rate with the 45.9% non-independence rate observed at 90 days underscores the reperfusion–outcome dissociation discussed in the main Discussion.*
